# Effect of L-arginine and L-Lysine HCl ratio on growth performance and ileum morphology of native chickens aged 2-14 weeks

**DOI:** 10.14202/vetworld.2022.1365-1372

**Published:** 2022-05-27

**Authors:** Charles Venirius Lisnahan, Oktovianus R. Nahak, Welsiliana Welsiliana, Lukas Pardosi

**Affiliations:** 1 Department of Animal Husbandry, Faculty of Agriculture, University of Timor, East Nusa Tenggara 85613, Indonesia; 2 Department of Biology, Faculty of Agriculture, University of Timor, East Nusa Tenggara 85613, Indonesia

**Keywords:** growth performance, ileum morphology, L-arginine, L-lysine HCl, native chickens

## Abstract

**Background and Aim:**

Micronutrients such as essential amino acids in chicken feed must be balanced to promote optimal development. The balance of the amino acids arginine and lysine in chicken feed is particularly important. This study aimed to examine the effect of the ratio of L-arginine to L-Lysine HCl on growth performance and ileum morphology of native chickens aged 2-14 weeks-old.

**Materials and Methods:**

One hundred and eighty 2-week-old native chickens which initial weight 78.10±4.97 g were classified into six treatments and five repetitions using a completely randomized design.

**Treatments were based on the ratio of arginine to lysine in the feed:**

T1 (0.50% L-arginine: 0.85% L-lysine HCl); T2 (0.75% L-arginine: 0.85% L-lysine HCl); T3 (1.00% L-arginine: 0.85% L-lysine HCl); T4 (0.50% L-arginine: 1.00% L-lysine HCl); T5 (0.75% L-arginine: 1.00% L-lysine HCl); and T6 (1.00% L-arginine: 1.00% L-lysine HCl).

**Results:**

Groups T3 and T6 had the highest feed consumption (42.06±0.29 and 42.78±0.72 g/bird/day, respectively), while Group T6 had the highest body weight and body weight gain rate (1505.60±103.20 kg/bird and 16.99±1.24 g/bird/day, respectively). Groups T3 and T6 also had the highest carcass weight (916.16±46.99 and 947.18±62.32 g/bird, respectively). The best feed conversion was seen for Groups T3, T5, and T6 (2.55±0.14, 2.50±0.20, and 2.53±0.19, respectively). For ileum morphometry, the highest villus height occurred in Groups T2, T3, T5, and T6 (962.80±23.31, 982.80±10.03, 972.80±18.99, and 989.80±10.69 μm, respectively); and Group T6 had the highest crypt depth and villus width (340.80±11.52 and 302.00±4.00 μm, respectively). Statistical analysis indicated significant differences among the treatment groups for all variables examined (p<0.05).

**Conclusion:**

The highest ratio of arginine-lysine was associated with the largest increase in native chicken feed consumption, body weight gain, feed conversion, and carcass weight, as well as villus height and width, and crypt depth in the ileum. Overall, an arginine-lysine ratio of 0.8-1.20 promoted optimal growth of native chickens aged 2-14 weeks. In the future, it is important to increase the arginine-lysine ratio with low feed protein levels in native chickens.

## Introduction

The majority (70%) of production costs for native chickens are associated with feed costs. Feeds containing fishmeal and soybean as protein sources are expensive and alternative protein sources such as synthetic amino acids could reduce production costs. Standard requirements for the essential amino acids methionine, lysine, threonine, and tryptophan for native chickens were previously reported [1,2]. Arginine is another amino acid required for optimal growth of poultry and serves as a nitric acid trigger that can enhance blood circulation to carry nutrients and stimulate growth [3]. Murakami *et* *al*. [4] demonstrated that arginine together with glycine and methionine can act as precursors for the production of creatine that is required for muscle growth.

Arginine, as well as cysteine, glutamine, leucine, proline, and tryptophan constitute functional amino acids that play key roles in metabolic pathways that are required for physiological maintenance, development, reproduction, and immunity [5]. Addition of arginine to feed can maximize feed efficiency and protein aggregation as well as decrease adiposity and improve overall livestock health. Toprak *et al*. [6] described arginine as one of the most multifunctional amino acids in animal cells. Indeed, arginine is required for the synthesis of several compounds, including ornithine, polyamine (spermidine, spermine, and putrescine), proline, creatine, protein, nitric oxide, and citrulline. Arginine also plays a role in enhancing insulin release, growth hormone, and insulin-like growth factor 1 within bloodstream [7].

Poultry does not have a urea cycle and thus cannot synthesize arginine and lysine. Arginine and lysine constitute essential amino acids for poultry, particularly during the starter and growth phases. For native chickens, arginine and lysine availability are crucial, again due to a lack of synthesized arginine and endogenous lysine, which are critical for rapid protein deposition and muscle formation during starter and growth phases. Lysine plays a significant role in producing protein in muscle and other body tissues. Together with Vitamin C, lysine is important for formation of collagen and other connective tissues (cartilage and joints). Lysine also contributes to increased intake of low protein feeds [8]. Mukhtar *et al*. [9] showed that the inclusion of amino acids in feed promotes increases in feed intake while reducing feed efficiency, decreasing nitrogen retention, and increasing uric acid excretion.

In poultry feed, arginine must be balanced with lysine. Feeds that had an excess of either arginine or lysine can have antagonistic effects on chickens, such as reduced growth [3]. Araujo *et al*. [10] found that excessive lysine substantially enhances arginine requirements, and the presence of a slight excess of lysine relative to arginine was associated with stunted growth [11].

Previous studies [3,11] demonstrated the importance of balanced arginine and lysine content in feed for broiler chickens, but the effects of this balance in native chickens are unclear. This study aimed to examine the effect of L-arginine and L-Lysine HCl ratio on growth performance and ileum morphology of native chickens aged 2-14 weeks.

## Materials and Methods

### Ethical approval

The study protocol was approved by the animal ethics committee of the Animal Science Study Program, Agriculture Faculty, Timor University, Indonesia.

### Study duration, location, animals, and feed preparation

The study was conducted from May to September 2021 in the Sasi Kefamenanu Sub-district of Indonesia. Laboratory analyses were performed in the Laboratory of Agriculture Faculty, Timor University, Laboratory of Nutrient and Animal feed of the Faculty of Animal Husbandry, as well as the Animal Health Laboratory of Nusa Cendana University, Kupang, Indonesia.

A total of 180 native chickens whose initial weight 78.10±4.97 g were housed in 30 1.0×1.0×0.7 m litter cages. Feed bins, water dispensers, and bulbs, weighing scale, and equipment for analysis of ileum morphometry were also used. The feed materials included yellow corn, rice bran, soybean meal, fishmeal, calcium carbonate, and vitamin premix, methionine, lysine, arginine, threonine, and tryptophan. Other materials included those needed to analyze ileum morphometry.

### Dietary treatment and feeding duration

This study used a completely randomized design using six treatments and five repetitions that comprised 6 native chickens (Table-1). Treatments groups are:

- T1: 0.50% L-arginine: 0.85% L-lysine HCl in feed (ratio 0.6)
- T2: 0.75% L-arginine: 0.85% L-lysine HCl in feed (ratio 0.9)
- T3: 1.00% L-arginine: 0.85% L-lysine HCl in feed (ratio 1.2)
- T4: 0.50% L-arginine: 1.00% L-lysine HCl in feed (ratio 0.5)
- T5: 0.75% L-arginine: 1.00% L-lysine HCl in feed (ratio 0.8)
- T6: 1.00% L-arginine: 1.00% L-lysine HCl in feed (ratio 1.0).

**Table 1:** Composition and content of nutrients in different native chicken treatments.

Feed material	Treatment					

	T1	T2	T3	T4	T5	T6
Yellow corn (%)	56.00	56.00	56.00	56.00	56.00	56.00
Rice bran (%)	21.00	20.60	20.50	20.85	21.50	20.35
Fishmeal (%)	9.00	9.00	9.00	9.00	9.00	9.00
Soybean meal (%)	9.50	9.50	9.50	9.50	9.50	9.50
Dl-methionine (%)	0.25	0.25	0.25	0.25	0.25	0.25
L-threonine (%)	0.80	0.80	0.80	0.80	0.80	0.80
L-tryptophan (%)	0.10	0.10	0.10	0.10	0.10	0.10
L-lysine HCl (%)	0.85	1.00	0.85	1.00	0.85	1.00
L-arginine (%)	0.50	0.75	1.00	0.50	0.75	1.00
Mineral premix (%)	0.30	0.30	0.30	0.30	0.30	0.30
Vitamin premix (%)	0.30	0.30	0.30	0.30	0.30	0.30
Calcium (%)	0.90	0.90	0.90	0.90	0.90	0.90
Phosphor (%)	0.40	0.40	0.40	0.40	0.40	0.40
Total (%)	100.00	100.00	100.00	100.00	100.00	100.00
Composition of nutrients						
Metabolized energy (kcal/kg)^1^	2983.02	2974.10	2971.88	2979.67	2994.16	2968.54
Crude protein (%)^1^	17.92	17.88	17.87	17.91	17.98	17.85
Crude fat (%)^1^	5.39	5.36	5.35	5.38	5.44	5.34
Ash (%)^1^	7.84	7.80	7.79	7.82	7.89	7.77
Crude fiber (%)^1^	9.63	9.57	9.55	9.60	9.73	9.53
Methionine (%)	0.32	0.32	0.32	0.32	0.32	0.32
Threonine (%)	1.00	1.00	1.00	1.00	1.00	1.00
Tryptophan (%)	0.27	0.27	0.27	0.27	0.27	0.27
Lysine (%)	0.92	1.07	0.92	1.07	0.92	1.07
Arginine (%)	0.68	0.93	1.18	0.68	0.93	1.18
Calcium (%)	1.84	1.84	1.84	1.84	1.84	1.84
Available phosphor (%)	0.60	0.60	0.60	0.60	0.60	0.60

1 Analysis result of Biochemical Laboratory of the Faculty of Animal Husbandry UGM Yogyakarta, 2017

### Ileum morphology measurement

Ileum morphology was examined under a microscope (Olympus Corporation Japan), with 40x. The villus height and width, as well as crypt depth of ileum from 14 week-old native chickens were measured.

#### Preparation of chicken intestine sample

Segments of small intestines were first isolated to obtain ileum tissue. Pieces (2 cm) of the ileum were collected and fixed by soaking in 10% buffered formalin for 24-48 h.

#### Hematoxylin-eosin staining

The fixed tissues were hydrated in an alcohol series (70, 80, 90, and 95%), with soaking in each solution for around 10 s. The samples were placed in xylol and then dipped into paraffin. Using a microtome, the paraffin-embedded samples were cut into thin strips that were mounted on glass slides.

#### Imaging

The prepared samples were observed at 10× magnification with Olympus BX 51 microscope equipped with Olympus DP 12 projector (Olympus Corporation). Morphology images were viewed on a JVC TMH 1750 C monitor. To identify appropriate ileum morphology, the samples were imaged to allow measurement of ileum features. Measurements were carried out in triplicate for each parameter.

#### Measurement of villus height, villus width, and crypt depth of Lieberkuhn

The measurements of villus height, villus width, and depth of Lieberkuhn crypt were done using a flat-screen computer with the Microsoft Office Picture Manager program at 40% magnification. At first, the standard size of micrometer (μm) was determined with the help of a computer, namely, how much the magnification value used or desired is converted into units of length (μm). The μm unit number obtained was then used as a standard in measuring the villus height, villus width, and the depth of the crypt displayed on the monitor screen.

### Data collection and analysis

In addition to the measurements for the ileum, data were collected for each treatment group for several parameters of native chickens, including feed intake, body weight gain, feed conversion, carcass weight, and percentage. Body weight gain is the difference between the final and initial body weights. Feed conversion is the feed intake divided by body weight gain. Carcass weight was obtained after subtracting the weight of blood, feathers, head and neck, feet, internal organs, and abdominal fat. The collected data were analyzed using analysis of variance (ANOVA) based on Completely Randomized Design and Duncan’s Test using IBM SPSS Statistics 22 (IBM Corp., NY, USA).

## Results

### Body weight gain

**Figure-1:**
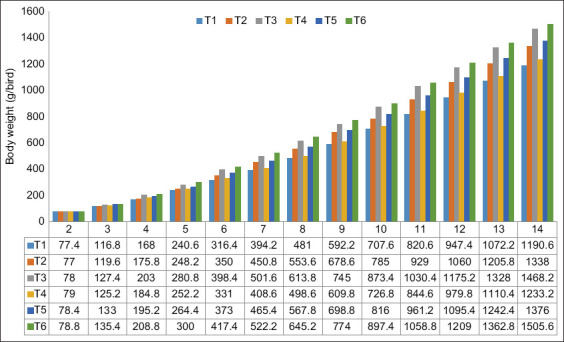
Body weight gain of native chickens during the study period.

The native chickens in the six treatment groups (T1-T6) were each fed the indicated diet for 12 weeks (2 weeks-old through 14 weeks-old; Table-2). At 14 weeks-old, chickens in T6 and T3, which received 1.0% L-lysine HCl in the feed, had the highest body weight among the groups (1505.60±103.20 g and 1468.20±74.88 g, respectively). Similarly, these two groups had the highest amount of body weight gain (16.99±1.24 g and 15.01±1.48 g). ANOVA showed that treatments involving arginine and lysine supplementation significantly impacted native chicken body weight gain (p<0.05). An arginine-lysine ratio of 0.8 (T2) increased body weight by 16.58% compared with an L-arginine level of 0.50% (T1). Body weight gains were observed as respective L-arginine and L-lysine levels increased to 1.00% and 0.85%. The increase was 9.13% compared with T2 (0.75% L-arginine). For T4, which received 1.00% L-lysine and 0.50% L-arginine, the body weight was significantly increased compared with T1 which had half the amount of L-arginine and nearly twice as much L-lysine. Meanwhile, T5 and T2 received feed with the same amount of L-arginine (0.75%), but different amounts of L-lysine (1.00% vs. 0.85%). The body weight was increased for T5 relative to T2. Overall, feed with the highest amount of both L-arginine and L-lysine (T6, 1.00% each) had the highest body weight of all the treatment groups (Figure-1).

**Table 2:** Native chicken performance aged 2-14 weeks.

Parameter	Treatments					

	T1	T2	T3	T4	T5	T6
Body weight (g)	1190.60±21.33^e^	1338.00±120.64^cd^	1468.20±74.88^ab^	1233.20±8.04^de^	1376.00±124.65^bc^	1505.60±103.20^a^
Body weight gain (g)	13.25±0.22^d^	15.01±1.48^bc^	16.55±0.91^a^	13.74±0.16^cd^	16.38±1.48^ab^	16.99±1.24^a^
Feed intake	38.21±0.78^d^	39.73±0.48^c^	42.06±0.29^a^	38.78±0.45^d^	40.79±0.71^b^	42.78±0.72^a^
Feed Conversion	2.88±0.08^a^	2.67±0.28^ab^	2.55±0.14^b^	2.82±0.03^a^	2.50±0.20^b^	2.53±0.19^b^
Carcass weight (g)	708.92±17.44^c^	810.24±67.85^b^	916.16±46.99^a^	738.19±6.76^c^	841.41±77.94^b^	947.18±62.32^a^
Carcass percentage	59.54±0.74^c^	60.59±0.71^b^	62.40±0.32^a^	59.86±0.42^c^	61.14±0.43^b^	62.93±0.27^a^
Villus height (mm)	918.40±28.25^b^	962.80±23.31^a^	982.80±10.03^a^	934.80±23.36^b^	972.80±18.99^a^	989.80±10.69^a^
Villus width (mm)	269.40±6.88^d^	282.00±5.05^c^	292.80±7.46^b^	276.60±7.09^cd^	291.40±4.50^b^	302.00±4.00^a^
Crypt depth (mm)	306.00±7.81^d^	317.40±5.94^bc^	322.80±3.70^b^	311.40±2.79^cd^	322.80±3.63^b^	340.80±11.52^a^

Remarks: Superscript was different on the average row showing a significant difference (p<0.05); T1 (0.50% L-arginine: 0.85% L-lysine); T2 (0.75% L-arginine: 0.85% L-lysine); T3 (1.00% L-arginine: 0.85% L-lysine); T4 (0.50% L-arginine: 1.00% L-lysine); T5 (0.75% L-arginine: 1.00% L-lysine); T6 (1.00% L-arginine: 1.00% L-lysine)

### Feed intake

**Figure-2:**
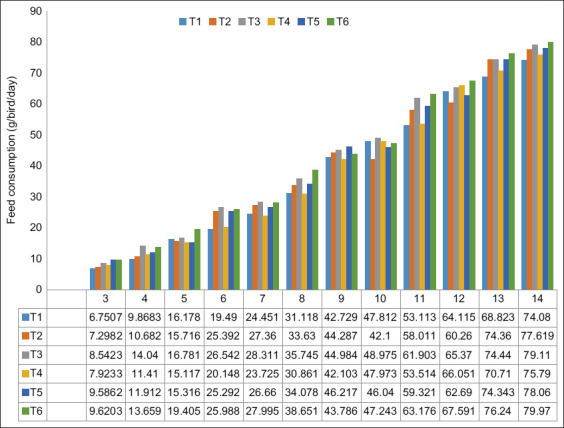
Feed consumption of native chickens over the study period.

As with body weight, T6 and T3 had the highest average feed consumption and the difference among the groups by ANOVA was significant (p<0.05; Table-2). Supplementation with 0.50% L-arginine and either 0.85% or 1.00% L-lysine (T2 and T4) did not affect feed consumption. However, when the level of L-arginine increased to 0.75% on 0.85% and was coupled with 1.00% L-lysine, feed consumption increased by 3.98% and 5.18%, respectively. Feed consumption further increased as the amount of L-arginine was increased to 1.00% with 0.85% or 1.00% L-lysine (Figure-2)

### Feed conversion ratio

**Figure-3:**
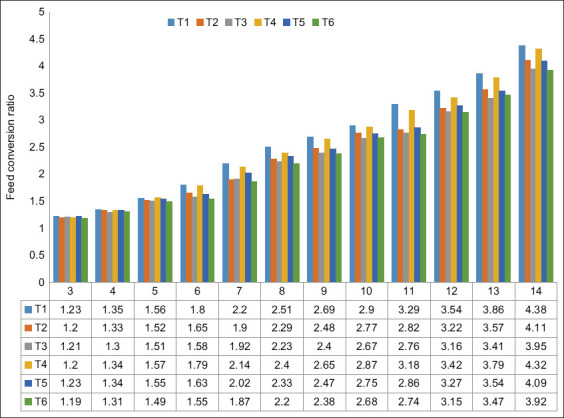
Native chicken feed conversion over the study period.

T1 and T4 had the highest feed conversion ratio, significantly differing from the other treatment groups (p<0.05). Both treatments had 0.50% L-arginine (Table-2). There was no significant difference between the ratios for the T1 and T4 groups. However, when the L-arginine level increased from 0.75% to 1.00%, the feed conversion decreased by 9.57% and 11.35% (Figure-3).

### Carcass weight

In addition to having the highest body weight and feed consumption rate, T6 and T3 had significantly higher (p<0.05) carcass weight than the other treatment groups. Meanwhile, supplementation with 0.50% L-arginine and either 0.85% (T1) or 1.00% L-lysine (T4) did not affect carcass weight. With feed having 0.75% L-arginine with 0.85% or 1.00% L-lysine (T2 and T4), the carcass weight increased by 9.76% and 13.98%. The carcass weight continuously increased for feed with 1.00% L-arginine (T3-T6; 8.88%-12.57% compared with T5). The carcass percentage similarly increased (2.06-2.93%).

### Ileal morphology

The average villus height in ileum of 14 week-old native chickens was significantly higher (p<0.05) for T6 compared to the other treatment groups (Table-2). Supplementation with 0.50% L-arginine did not impact villus height but increased to 0.75 and 1.00% L-arginine together with 0.85% or 1.00% L-lysine (T2 and T3, T5, and T6) did result in increased villus height (2.99 5.88%).

Chickens in T6 also had the largest villus width, and this difference was significant (p<0.05) relative to the other groups (Table-2). Supplementation with 0.50% L-arginine or with 1.00% L-lysine (T4) did not affect villus width, but increasing the L-arginine content to 0.75% (T2) resulted in a 4.68% villus width increase relative to T1. The villus width continuously increased for 1.00% L-arginine with 0.85% (T3) and 1.00 (T6) L-lysine (3.83% and 7.09%).

The average crypt depth was highest for T6 (Table-2) and ANOVA showed that the treatments significantly impacted this feature (p<0.05) as reflected by increases of around 5.5% relative to the next highest groups (T3 and T5). Supplementation with 0.50% L-arginine and 1.00% L-lysine (T4) did not affect depth (p<0.05). However, when the level of L-arginine increased to 0.75% (T2), the crypt depth increased by 3.73%. Similar increases (3.66%) were seen for 0.75% L-arginine with either 0.85% or 1.00% L-lysine (T3 and T5).

## Discussion

This study examined how varying amounts of arginine and lysine in feed affected feed consumption, body weight and other parameters of native chickens. Feed consumption decreased when the ratio of the two amino acids was 0.6 (T1) or 0.75 (T4). Meanwhile, when the L-arginine and L-lysine ratio was 0.8-1.0, the feed consumption increased. The average native chicken feed consumption was 42.78 g/bird/day.

Zampiga *et al*. [11] reported a correlation between arginine and lysine usage in increasing feed consumption and body weight of broilers. They also showed that increases in lysine improved arginine requirements and vice versa. When concentrations of either lysine or arginine were low, antagonism was observed [12]. Lisnahan and Nahak [2] reported that consumption of feed supplemented with tryptophan and threonine (without L-arginine and L-lysine) was 124.18 g/bird/week for native chickens in the starter phase (i.e., 1-6 weeks), whereas consumption in the grower phase (aged 7-14 weeks) was lower at 50.10 g/bird/day.

Arginine plays a crucial role in various metabolic, pathophysiological, and immunological processes in poultry, which lack a functional urea cycle [11]. native chickens cannot synthesize arginine, and thus for poultry arginine is an essential amino acid. Chickens exclusively depend on dietary arginine sources; therefore, adequate amounts should be given in the feed.

Body weight correlates with feed consumption. In this study, body weight increased as the arginine and lysine ratio increased, and the observed increase was significant for the 0.8-1.2 ratio compared with the 0.6-0.75 ratio. This result indicated a correlation between arginine and lysine to native chicken body weight. Deficiency or excess of each amino acid had a negative effect on amino acid concentrations in plasma and muscle as well as on growth performance [13]. This effect was more evident when lysine is in excess (i.e., low arginine: lysine ratio) compared to when arginine is in excess (i.e., high arginine: lysine ratio) [14]. Excess dietary lysine was reported to have no effect on digestibility or arginine absorption but did inhibit kidney reabsorption and stimulate kidney arginase activity [13]. According to the NRC [15], the optimum arginine-lysine ratio for broilers should be 1.14 for the first 3 weeks, 1.10 from week 3 to week 6, and 1.18 from week 6 to week 8. Based on the previous studies, Balnave and Brake [13] suggested that the optimum arginine-lysine ratio should be between 0.90 and 1.18. Another report suggested ratios of 1.05 and 1.08 for 0-21 days and 21-42 days, respectively [16].

Feed conversion ratios compare feed consumed with body weight gain. A low feed conversion ratio indicates better feed efficiency. Here, an arginine-lysine ratio of 0.80-1.2 produced better feed conversion ratios than the 0.6-0.75 ratio. This result is consistent with a report by Barekatain *et al*. [17], which showed that a high arginine-lysine ratio produced the best feed efficiency for broilers. Labadan *et al*. [18] suggested that higher amounts of arginine are needed for feed conversion compared to live weight gain of broilers. Subsequently, they showed that live weight gain was optimized at 1.24% arginine, whereas feed conversion was optimized at 1.31%. They hypothesized that arginine usage efficiency in poultry decreases linearly with increasing lysine levels.

Breast and thigh muscle represent the largest proportion of native chicken carcasses. Breast muscle is highly dependent on feed consumption and particularly amino acid balance. Here, the arginine-lysine ratio significantly affected native chicken carcass weight. For the 0.6-0.8 arginine-lysine ratio, the carcass weight was lower than that for the 1.0-1.2 ratio. This result indicated that dietary arginine can substantially affect chicken carcass components. Wu [16] reported that the amino acid composition in the body of 10 day-old broiler chicks had 111% higher arginine content than lysine. Based on this result, he indicated that arginine content of feeds for native chickens should be increased, particularly since L-arginine could be metabolized into citrulline and nitric oxide by nitric oxide synthase [19,20]. Nitric oxide has significant vasodilator activity [19] and can improve blood circulation to muscles, especially those in the breast and thigh. Fouad *et al*. [21] reported that breast muscle thickness can indeed be increased significantly by increasing the feed arginine level.

If the lysine level in feed is high and the level of arginine is low, then arginase activity in the kidneys increases, and, in turn, arginine degradation increases [3,4]. As a result, less arginine is available for protein synthesis and arginine-dependent biological functions. Excess lysine also lowers activity of arginine-lysine aminotransferase [10,11], which catalyzes the first stage of creatine synthesis and subsequently decreases creatine concentrations in muscle [19]. The antagonism between arginine and lysine is more evident in poultry that lack carbamoyl phosphate synthetase I and thus cannot synthesize arginine. Poultry also has lower activity of argininosuccinate synthetase, argininosuccinate lyase, and ornithine transcarbamylase in the kidneys [11]. Therefore, arginine is an essential amino acid for poultry and must be supplemented in the feed to support growth.

In our study, native chickens given feed supplemented with balanced and high amounts of L-arginine and L-lysine (T6) had improved intestinal morphology as evidenced by higher and wider villi and deeper crypts. These morphometric improvements translated to enhanced feed intake and a feed conversion ratio that reflected more efficient feed utilization. Maiorka *et al*. [22] showed that livestock with increased intestinal mucosa cell regeneration has more villi that arise from enhanced mitosis and hyperplasia activities. Intestinal mucosa grows continuously due to cell desquamation in the intestinal lumen, and cell regeneration can be promoted by increased feed intake. Here, a positive effect of the arginine-lysine ratio on ileum mucosa morphology was observed in 14 week-old native chickens and there was a correlation between growth rate and feed consumption.

Moreover, as the growth rate increased, ileum morphology was enhanced to promote nutrient absorption. Arginine and lysine have been shown to be involved in maintaining intestinal mucosa and this effect can be associated with better performance of native chickens. Consistent with this finding, Lisnahan *et al*. [8] reported that villus height, crypt depth, and villus width contribute to the intestinal mucosa area available for digestion and nutrient absorption for use in the development and maintenance of body tissues.

There was a relationship between villi height and ileal crypt depth of native chickens (Table-2). An increase in villous height and crypt depth was associated with more villous surface area to absorb nutrients into the bloodstream [23]. On the other hand, it was also stated that the ratio of villi height to crypt depth indicated an increase in the area for nutrient absorption [24,25]. Likewise, Lisnahan and Nahak [2] in more detail stated that the increase in villi height and crypt depth in the ileum of native chickens was parallel to the increase in digestive and absorption functions due to the expansion of the absorption area and is an expression of the nutrient transport system throughout the body.

Villus height also likely has a positive relationship with body weight gain as well as feed consumption and feed efficiency [2]. The regulatory mechanism exerted on the intestinal mucosa remains poorly understood. There is evidence that amino acids could affect the interplay between intrinsic afferent neurons and primary extrinsic neurons, as well as the central nervous system by regulating transcription and expression of genes involved in amino acid metabolism, as has been reported for several amino acids, including arginine and lysine [16,26]. Arginine is involved in synthesis of polyamines that are related to cell division, protein synthesis, and tissue growth [24], as well as intestinal growth [23,26]. Polyamine levels in poultry are highly responsive to manipulation of arginine concentrations. Again, since poultry lack a functional area cycle, exogenous arginine is needed to form ornithine, which, in mammals, is generated from glutamate acid [27]. Murakami *et al*. [4] showed that decreased polyamine levels inhibit proliferation, migration, and apoptosis of intestinal cells. As a polyamine precursor, arginine can act as a trophic agent that stimulates the growth of intestinal mucosa by accelerating mitosis in the villus-crypt area. Such activity increases the number and size of villus cells [28]. Wu [5], Barekatain *et al*. [17], and Ball *et al*. [29] found that amino acids induce gene transcription by activating important enzymes involved in intestinal mitosis, such as glutamine in ornithine-decarboxylase, an enzyme that mediates ornithine decarboxylation in polyamine synthesis. Amino acids also modulate gene expression through specific processes that entail information transfer from the coding gene to the gene products (e.g., RNA and/or protein) [23,29]. Putrescine is formed from ornithine by spermine decarboxylase, as well as from spermidine obtained from putrescine arising from decarboxylation of S-adenosyl methionine, which is derived from methionine [9,30]. This biogenic amine is an important factor in the growth and development of small intestinal mucosa [11,25,31].

## Conclusion

A high arginine-lysine ratio increases feed consumption, body weight gain, feed conversion, carcass weight, villus height, crypt depth, and ileum villus width of native chickens. An arginine-lysine ratio of 0.8-1.2 was associated with optimal growth of native chickens between 2 and 14 weeks-old. In the future, it is important to increase the arginine-lysine ratio with low feed protein levels in native chickens.

## Authors’ Contributions

CVL: Conceived ideas and designed the study, collected the data, supervised the study, analyzed the data, and drafted the manuscript. ORN, WW and LP: Designed the study, feed preparation, laboratory work, and supervised the study. CVL, ORN, WW, and LP: Revised the manuscript. All authors read and approved the final manuscript.

## References

[1] Lisnahan C.V, Wihandoyo Zuprizal, Harimurti S (2017). Effect of addition of methionine and lysine into diets based on cafeteria stabdars on the growth performance of native chickens at starter phase. Int. J. Poult. Sci.

[2] Lisnahan C.V, Nahak O.R (2020). Growth performance and small intestinal morphology of native chickens after feed supplementation with tryptophan and threonine during the starter phase. Vet. World.

[3] Leeson S, Summers J. D (2008). Commercial Poultry Nutrition.

[4] Murakami A.E, Fernandes J.I.M, Hernandes L, Santos T.C (2012). Effects of starter diet supplementation with arginine on broiler production performance and on small intestine morphometry. Pesq. Vet. Bras.

[5] Wu G (2009). Amino acids:Metabolism, functions, and nutrition. Amino Acids.

[6] Toprak N.N, Yavaş I, Çenesiz A.A, Ceylan N, Çiftci I (2021). Effects of digestible amino acid-based formulation of low protein broiler diets supplemented with valine, isoleucine and arginine on performance and protein efficiency. Czech J. Anim. Sci.

[7] Newsholme P, Brennnan L, Rubi B, Maechler P (2005). New insights into amino acid metabolism, beta-cell function and diabetes. Clin. Sci.

[8] Lisnahan C.V, Wihandoyo Zuprizal, Harimurti S (2017). Growth performance of native chickens in the grower phase fed methionine and lysine-supplemented cafeteria standard feed. Pak. J. Nutr.

[9] Mukhtar M.A, Makkawi A, Tigani M (2010). Effect of amino acids supplementation to marginally deficient local broiler chick diets. J. Sci. Technol.

[10] Araujo L.F, Junquera O.M, Araujo C.S.S, Barbosa L.C.G, Ortolan J.H, Faria D.E, Stringhini J.H (2005). Energy and lysine for broilers from 44 to 55 days of age. Braz. J. Poult. Sci.

[11] Zampiga M, Laghi L, Petracci M, Zhu C, Meluzzi A, Dridi S, Sirr F (2018). Effect of dietary arginine to lysine ratios on productive performance, meat quality, plasma and muscle metabolomics profile in fast-growing broiler chickens. J. Anim. Sci. Biotechnol.

[12] Abdolalizadeh A.F, Ebrahimi M, Daghigh K.H (2017). Effect of *in ovo* injection of different ratios of L-arginine to L-lysine on body growth, muscle production, and blood metabolites concentration of day-old Ross broiler chicks. Iran. J. Anim. Sci.

[13] Balnave D, Brake J (2002). Re-evaluation of the classical dietary arginine:Lysine interaction for modern poultry diets:A review. Worlds Poult. Sci. J.

[14] Sirathonpong O, Ruangpanit Y, Songserm O, Koo E.J, Attamangkune S (2019). Determination of the optimum arginine:Lysine ratio in broiler diets. Anim. Prod. Sci.

[15] NRC (1994). National Research Council:Nutrient Requirement of Poultry.

[16] Wu G (2014). Dietary requirements of synthesizable amino acids by animals:A paradigm shift in protein nutrition. J. Anim. Sci. Biotechnol.

[17] Barekatain R, Nattrass G, Tilbrook A, Chousalkar K, Gilani S (2019). Reduced protein diet and amino acid concentration alter intestinal barrier function and performance of broiler chickens with or without synthetic glucocorticoid. Poult. Sci.

[18] Labadan M.C, Hsu K.N, Austic R.E (2001). Lysine and arginine requirements of broiler chickens at two to three weeks intervals to eight weeks of age. Poult. Sci.

[19] Fernandes J.I.M, Murakami A.E (2010). Arginine metabolism in uricotelic species. Acta Sci. Anim. Sci.

[20] Khajali F, Wideman R.F (2010). Dietary arginine:Metabolic, environmental, immunological and physiological interrelationships. Worlds Poult. Sci. J.

[21] Fouad A.M, El-Senousey H.K, Yang X.J, Yao J.H (2012). Role of dietary L-arginine in poultry production. Int. J. Poult. Sci.

[22] Maiorka A, Santin E, Dahlke F, Boleli I.C, Furlan R.L, Macari M (2003). Post hatching water and feed deprivation affect the gastrointestinal tract and intestinal mucosa development of broiler chicks. J. Appl. Poult. Res.

[23] Monavvar H.G, Moghaddam G, Ebrahimi M (2020). Effect of arginine on growth performance, meat quality, intestine morphology, and immune system of broiler chickens. A review. Iran. J. Appl. Anim. Sci.

[24] Yu J, Yang H, Wang Z, Dai H, Xu L, Ling C (2018). Effects of arginine on the growth performance, hormones, digestive organ development and intestinal morphology in the early growth stage of layer chickens. Ital. J. Anim. Sci.

[25] Abdulkarimi R, Shahir M.H, Daneshyar M (2019). Effects of dietary glutamine and arginine supplementation on performance, intestinal morphology and ascites mortality in broiler chickens reared under cold environment. Asian Australas J. Anim. Sci.

[26] Vicuna E, Kuttappan V, Galarza-Seeber R, Latorre J, Faulkner O, Hargis B, Tellez G, Bielke L (2015). Effect of dexamethasone in feed on intestinal permeability, differential white blood cell counts, and immune organs in broiler chicks. Poult. Sci.

[27] Rhoads J.M, Wu G (2009). Glutamine, arginine, and leucine signaling in the intestine. Amino Acids.

[28] Tan J, Applegate T.J, Liu S, Guo Y, Eicher S.D (2014). Supplemental dietary L-arginine attenuates intestinal mucosal disruption during a coccidial vaccine challenge in broiler chickens. Br. J. Nutr.

[29] Ball R.O, Urschel K.L, Pencharz P.B (2007). Nutritional consequences of interspecies differences in arginine and lysine metabolism. J. Nutr.

[30] Barekatain R, Chalvon-Demersay T, McLaughlan C, Lambert W (2021). Intestinal barrier function and performance of broiler chickens fed additional arginine, a combination of arginine and glutamine or an amino acid-based solution. Animals (Basel).

[31] Adibmoradi M, Ebrahimi M, Zare S.A, Shivazad M, Ansari P.Z, Tebianian M, Nourijelyani K (2015). The effects of L-arginine on growth, small intestine, and immune system of broilers in starter period. Iranian J. Anim. Sci.

